# Habitat Heterogeneity Determines Climate Impact on Zooplankton Community Structure and Dynamics

**DOI:** 10.1371/journal.pone.0090875

**Published:** 2014-03-10

**Authors:** Saskia A. Otto, Rabea Diekmann, Juha Flinkman, Georgs Kornilovs, Christian Möllmann

**Affiliations:** 1 Stockholm Resilience Centre, Stockholm University, Stockholm, Sweden; 2 Institute for Hydrobiology and Fisheries Science, Center for Earth System Research and Sustainability (CEN), KlimaCampus, University of Hamburg, Hamburg, Germany; 3 Marine Research Centre, Finnish Environment Institute SYKE, Helsinki, Finland; 4 Department of Fish Resources Research, Institute of Food Safety, Animal Health and Environment, Riga, Latvia; University of Hamburg, Germany

## Abstract

Understanding and predicting species distribution in space and time and consequently community structure and dynamics is an important issue in ecology, and particularly in climate change research. A crucial factor determining the composition and dynamics of animal populations is habitat heterogeneity, i.e., the number of structural elements in a given locality. In the marine pelagic environment habitat heterogeneity is represented by the distribution of physical oceanographic parameters such as temperature, salinity and oxygen that are closely linked to atmospheric conditions. Little attention has been given, however, to the role of habitat heterogeneity in modulating the response of animal communities to external climate forcing. Here we investigate the long-term dynamics of *Acartia* spp., *Temora longicornis*, and *Pseudocalanus acuspes*, three dominant zooplankton species inhabiting different pelagic habitats in the Central Baltic Sea (CBS). We use the three copepods as indicator species for changes in the CBS zooplankton community and apply non-linear statistical modeling techniques to compare spatial population trends and to identify their drivers. We demonstrate that effects of climate variability and change depend strongly on species-specific habitat utilization, being more direct and pronounced at the upper water layer. We propose that the differential functional response to climate-related drivers in relation to strong habitat segregation is due to alterations of the species’ environmental niches. We stress the importance of understanding how anticipated climate change will affect ecological niches and habitats in order to project spatio-temporal changes in species abundance and distribution.

## Introduction

Understanding and predicting species distribution in space and time and consequently community structure and dynamics is an important issue in ecology, and particularly in climate change research. Animal populations in terrestrial and aquatic environments shift their geographical range or show strong fluctuations in abundance in response to climate-induced changes in, e.g., temperature, precipitation or ocean circulation [1]–[5]. Changes on the individual species and population level can eventually cause community re-organizations on large spatial scales [6]–[9] with concomitant effects on ecosystem functioning [10].

A crucial factor determining the composition and dynamics of animal populations is habitat heterogeneity, i.e., the number of structural elements in a given locality [11], [12]. For instance, species diversity is known to increase with habitat heterogeneity [13]–[16], since structurally diverse habitats promote the coexistence of a higher number of species by providing more niches and ways of exploiting environmental resources [17], [18]. In aquatic systems, mostly submerged plants and substrate material determine the physical structure of the environment, and therefore, have a considerable influence on the distributions and interactions of demersal fish species [14], [19], seagrass, stream or sub- and intertidal macroinvertebrates [20]–[23]. In the marine pelagic environment however, habitat heterogeneity is represented by the distribution of physical oceanographic parameters such as temperature, salinity and oxygen. Although these hydro-climatic variables are closely tied to atmospheric conditions, the question of how habitat heterogeneity modulates the response of animal communities to external climate forcing has received little attention so far.

**Figure 1:**
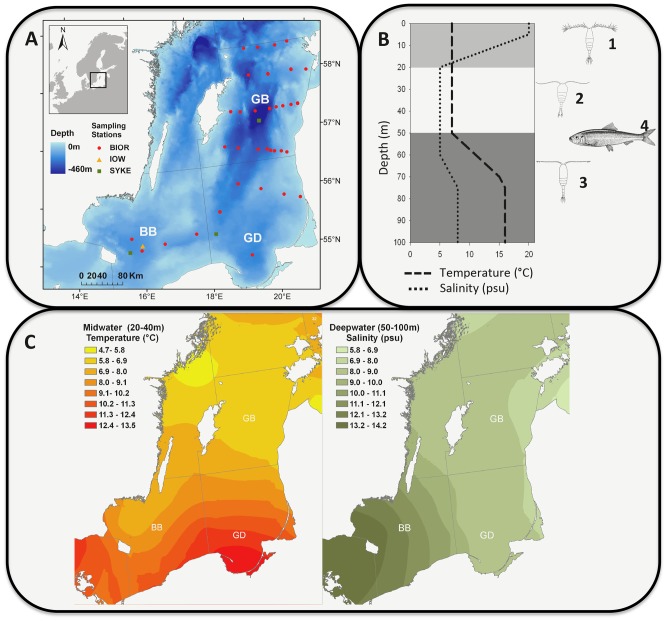
Study area. (A) Map of the Central Baltic Sea with its three basins (BB = Bornholm Basin, GD = Gdansk Deep, GB = Gotland Basin) including the sampling station of the different datasets. (B) Schematic vertical profile of the hydrology together with the copepod (1 = *Acartia* spp., 2 = *Temora longicornis*, 3 = *Pseudocalanus acuspes*) and predator distribution (4). (C) Horizontal profile of the hydrology in August 2001, i.e., the gradient in temperature and salinity at different water depths. The respective depth ranges were chosen based on the species most affected by these parameters in our analysis.

The pelagic of the Central Baltic Sea (CBS) is characterized by spatial hydrographic gradients that create a number of different habitats for local animal populations. Horizontally, both salinity and temperature generally decline from West to North-East (Fig. 1C). Even more pronounced, vertical habitat heterogeneity is induced by stratification of the water column during most of the productive season into (i) a warm but low saline surface layer, (ii) a cold and low saline intermediate layer, and (iii) a cold and highly saline deep layer (Fig. 1B). Here we investigate the importance of the spatial habitat structure for the Baltic Sea zooplankton community. Zooplankton is a suitable indicator of the effect of climate change on aquatic ecosystems and long-term changes in marine zooplankton are well studied, e.g. [24]–[26].

The CBS zooplankton community is composed of six major taxonomic groups (copepods, cladocerans, rotifers, appendicularian, ctenophores, polychaetes, and bivalves), dominated by very few species (often 1 to 3) [27], [28]. Physiological adaptations to physical oceanographic conditions result in different utilization of the different water masses or habitats available [27], [28]. In our study we investigate the long-term dynamics of *Acartia* spp., *Temora longicornis*, and *Pseudocalanus acuspes*, three dominant zooplankton species that make up to 60% of the spring biomass in the CBS and distribute differently according to these habitats. *Acartia* spp. mainly inhabits the surface water, *T. longicornis* the mid-water and *P. acuspes* the deeper, saline layer (Fig. 1B) [29], [30]. The vulnerability of our target species to predation is as well strongly habitat-specific. The dominant planktivore predators herring (*Clupea harengus*) and sprat (*Sprattus sprattus*) feed mainly in deeper waters [31], hence are displaying a different overlap with their zooplanktonic prey species (Fig. 1B).

Here we use the three copepods as indicator species for changes in the CBS zooplankton community, and specifically test (i) whether our target zooplankton species display different population trajectories in space and time, and (ii) if hydro-climatic factors and predation pressure differentially affect the long-term development of the individual specieś populations. We apply statistical modeling techniques to compare species-, area-, and season-specific long-term population trends and to identify their main drivers.

## Materials and Methods

### Zooplankton data

For our study we have, for the first time, combined data sets from three long-term zooplankton monitoring programs in the Central Baltic Sea (CBS; Fig. 1A). With this new and unique data set we were able to investigate the CBS zooplankton dynamics within all three major basins of the CBS (the Bornholm Basin (BB), Gdansk Deep (GD), and Gotland Basin (GB)) for the recent five decades (1960–2008). The data sets were provided by the Latvian Institute of Food Safety, Animal Health and Environment (BIOR), the Finnish Environment Institute (SYKE), and the Leibniz-Institute for Baltic Sea Research (IOW) in Germany. The programs differ in gear type and spatio-temporal coverage, with the Latvian program being the most comprehensive and described in detail by Möllmann *et al.* [25]. For an overview of sampling programs see Table S1.

In order to construct basin-specific time series on spring (sampling mainly in May) and summer (sampling mainly in August) biomass (mg*m^−3^) for *Acartia* spp., *Temora longicornis* and *Pseudocalanus acuspses*, we merged the three data sets according to the following procedure:

- We started by calculating the annual mean basin- and season-specific biomass of each species for each data set.
- After log10(x+0.001) transformation, we combined the three data sets by calculating annual means for each species, basin and season weighted by the sampling number of each individual sampling program.
- Subsequently, we accounted for differences between sampling programs due to gear-specific capture efficiencies and different spatial and temporal resolutions following the approach suggested by Mackas and Beaugrand [24] and computed species-specific log10(x+0.001) transformed anomalies:
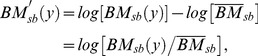
(1)where *BM_sb_*(y) is the mean biomass of a species in season s and basin b in a particular year y, 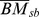 represents the season- and basin-specific overall time series mean for each species.

Using log-transformed anomalies accounts for any potential sampling bias that is inherent in each annual mean value as well as in the overall time series mean [24]. It should, however, be noted that this approach does not allow for a quantitative comparison between basins, seasons, or species, but rather provides a basis for comparisons of long-term trends.

### Environmental covariates

We included several hydro-climatic variables and predation pressure as covariates in our statistical analyses. The main hydrographic variables affecting *Acartia* spp., *T. longicornis* and *P. acuspes* are water temperature and salinity [25], [32]–[34]. We computed therefore basin-specific time series of seasonal (spring – April/May; summer – July/August) temperature and salinity conditions (Fig. S1). For both time periods, we calculated mean values of individual depth ranges inhabited by each copepod species based on [29], [30], [33], [35], including 0 to 20 m and 20 to 40 m for *Acartia* spp. and *T. longicornis* respectively (see Table S2). *P. acuspes* displays a strong ontogenetic shift in depth distribution (e.g., nauplii and copepodites C1-3 dwelling in the upper and mid-waters; copepodites C4-5 and adults dwelling close to or in the deep permanent halocline) and characteristic stage-distributions in each season [33], [36]. Hence, we considered three different depth ranges for spring, i.e., 20–60 m for early copepodites and 60–90 m (BB/GD) or 70–100 m (GB) for older stages, depending on the local depth of the permanent halocline. Eventually, we assumed 30–50 m to be the distribution range of the *P. acuspes* population in summer. Hydrographic data were retrieved from the International Council for the Exploration of the Sea (ICES) website (http://www.ices.dk/ocean/aspx/HydChem/HydChem.aspx).

As an indicator for the overall climate condition we used the Baltic Sea Index (BSI) provided by the GEOMAR Helmholtz Centre for Ocean Research Kiel, Germany (Table S2, Fig. S2A). This local climate mode represents the difference of normalized sea level pressure anomalies between Oslo in Norway and Szcecin in Poland. Although closely correlated to the North Atlantic Oscillation (NAO), the BSI generally has been better related to local oceanographic processes in the Baltic than remote, large-scale atmospheric patterns such as the NAO [37]. We used the winter BSI (December-March) since winter patterns of local and regional wind fields have a strong influence on the hydrographic situation in early spring [38], affecting phytoplankton dynamics and the onset of the spring bloom [39].

Sprat (*Sprattus sprattus*) is the dominant planktivore in the Baltic Sea basins and, at times, appears capable of exerting top-down control of *P. acuspes* population [40]. This makes sprat stock size generally a suitable indicator of predation pressure [41]. However, official stock size estimates for sprat are not available prior to 1974 [42]. Hence, a predation index (PI) was developed using stock size estimates for cod (*Gadus morhua*). Eastern Baltic cod is the major piscivore in the Baltic Sea and its stock size is inversely related to the stock size of sprat [41], [43] (Fig. S2B). We used spawning stock biomass (SSB) estimates for cod from official stock estimates that date back to 1966 [42], and extended the time series back to 1960 using estimates by Eero *et al*. [44]. The combined time series was inverted to mimic the predation pressure by planktivorous sprat and standardized to values between 0 and 1 and. The max. value of cod SSB is represented therefore by a PI value of 0 while the min. cod SSB value is indicated by a PI value of 1.

Food-limitation can be an important influence on population dynamics of copepod species, but studies on the feeding ecology of Baltic copepods in the field are scarce. Some earlier studies indicate a stronger importance of hydro-climatic variables as well as predation for the dynamics of *P. acuspes* [41], [43]. Other recent studies show the importance of food availability for the dynamics of *Acartia longiremis*. and *T. longicornis* in different seasons [35], [45]. Unfortunately, no reliable estimates of the different food sources were available for our full investigation period. Changes in phytoplankton abundances and timing are often affected by temperature, e.g., through changes in light-saturated photosynthesis rates [46], as well as by irradiance (cloud/solar radiation) and wind-induced vertical mixing intensity [47], [48]. Mixing intensity in particular is a key variable for growth performance as it determines largely the light and nutrient conditions [49]. Under westerly wind conditions (represented by positive BSI anomalies), cloudiness is generally higher and wind mixing reduced [37], [39]. We therefore assumed that dynamics of food availability are indirectly reflected by the temperature and BSI time series.

### Statistical analysis

We used Generalized Additive Modeling (GAM) techniques [50], [51] in this analysis which have the advantage of not requiring an a priori specification of the relationship between the response variable (Y) and the explanatory variable (X). Each Y_i_ is here linked with X_i_ by a smoothing function instead of a coefficient β as in traditional regression techniques and hence relationships do not have to be linear.

In a first analysis we tested for spatial differences in temporal trends in species biomass between the three basins. We modeled the log-transformed spring or summer biomass anomalies of each species either as a single function of time (year), i.e., for the entire CBS area,
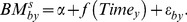
(2)or as basin-specific functions of time
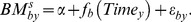
(3)where 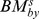 is the season-specific (index s) log-transformed biomass anomaly in basin b and year y. *f* and *f_b_* are the single and respectively basin-specific thin plate regression spline functions of the variable time. α is the intercept and ε_by_ the random noise terms assumed to be normally distributed with zero mean and finite variance. To focus only on the major trends and avoid over-parameterization, we limited the effective degrees of freedom (edf) to a maximum of 3. For each of the species- and season-specific comparisons between models including a spatially uniform or basin-specific smoothing functions, we applied an *F*-ratio test [51]. Significant differences (p-values<0.05) indicated here that the more complex GAMs with basin-specific smoothers performed better and, hence, long-term trends are better described for each basin separately.

In a second analysis we used the non-linear regression technique to identify the role of climate, hydrography and predation pressure on the seasonal long-term trends of each copepod. Each species' spring and summer biomass was then modeled by basin-specific smoothing functions of the different environmental variables, independent of whether long-term trend were significantly different between basins. The following model was used:
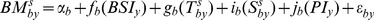
(4)with α_b_ as the intercept at basin b and *f_b_*, *g_b_*, *i_b_*, and *j_b_* as basin-specific thin plate regression spline functions (maximum edf  =  4) describing the effect of the locale climate index BSI, depth-specific temperature (T) and salinity (S), and predation (PI) respectively. Prior to the analysis we tested for collinearity among the covariates using Pearson correlation coefficient and a variance inflation factor (VIF) of 3 as exclusion criterion [52]. In deciding which smoothing term to include in the final model, we applied a backward stepwise selection approach. Initially, we started with a full model that included a basin effect and basin-specific smoothers for all four covariates. As selection criterion we used the Akaike’s Information Criterion (AIC) [53]. The underlying statistical assumptions were then tested through residual diagnostics of the optimal model. For the selected spring model of *Acartia* spp. residuals showed temporal autocorrelation of lag 2, hence we extended the GAM to a Generalized Additive Mixed Model (GAMM) by including a correlation structure. Such an extension allows a more complex stochastic model structure and implies that the single elements of the response variable are not independent anymore [51].We tested various structures in the full model and chose the one performing best based on the AIC [54]. We then applied again a model selection routine. All analyses were performed using the package ‘mgcv’ (with version R2.10) [51] within the ‘R’ statistical and programming environment [55].

## Results

### Long-term trends

To understand how habitat heterogeneity affects the influence of climatic variability on zooplankton community structure and dynamics, we first examined the long-term development of our three indicator species *Acartia* spp., *Temora longicornis*, and *Pseudocalanus acuspes* in the Central Baltic Sea (CBS). We found *T. longicornis* and *P. acuspes* long-term trends to be spatially homogeneous, while long-term dynamics of *Acartia* spp. significantly differed between the three basins (Table 1, Table S3).

**Table 1:** Summary of the F-ratio tests comparing species- and season-specific long-term trends modeled across basins or for each basin separately.

Species-Season	Model	Residuals	df Residuals	Deviance	df Deviance	F statistic	P-value
*Acartia* spp. - Spring	*f*(Year)	121.35	16.15				
	*f*_basin_(Year)	118.06	14.04	3.29	2.11	5.40	0.001
*Acartia* spp. - Summer	*f*(Year)	112.16	9.40				
	*f*_basin_(Year)	108.72	8.06	3.45	1.33	5.21	0.001
*T. longicornis* - Spring	*f*(Year)	121.13	19.54				
	*f*_basin_(Year)	117.78	19.14	3.35	0.40	0.73	0.6
*T. longicornis* - Summer	*f*(Year)	112.94	17.02				
	*f*_basin_(Year)	109.61	16.41	3.32	0.61	1.23	0.3
*P. acuspes* - Spring	*f*(Year)	121.08	7.24				
	*f*_basin_(Year)	117.72	7.16	3.36	0.09	0.42	0.8
*P. acuspes* - Summer	*f*(Year)	112.03	4.87				
	*f*_basin_(Year)	106.21	4.35	5.81	0.52	2.19	0.1

Generalized Additive Models (GAM) with a single year-smoother *f*(Year) for the entire Central Baltic Sea and basin-specific smoothers *f*_basin_(Year) using the F-ratio tests for each species and season. The residual deviance and the residual degrees of freedom (df) are given for each model together with the reduction in deviance and the change in df's, the F-statistic and its probability value. P-values<0.05 (in bold) indicate a better performance of the more complex GAM with basin-specific smoothers.

**Figure 2:**
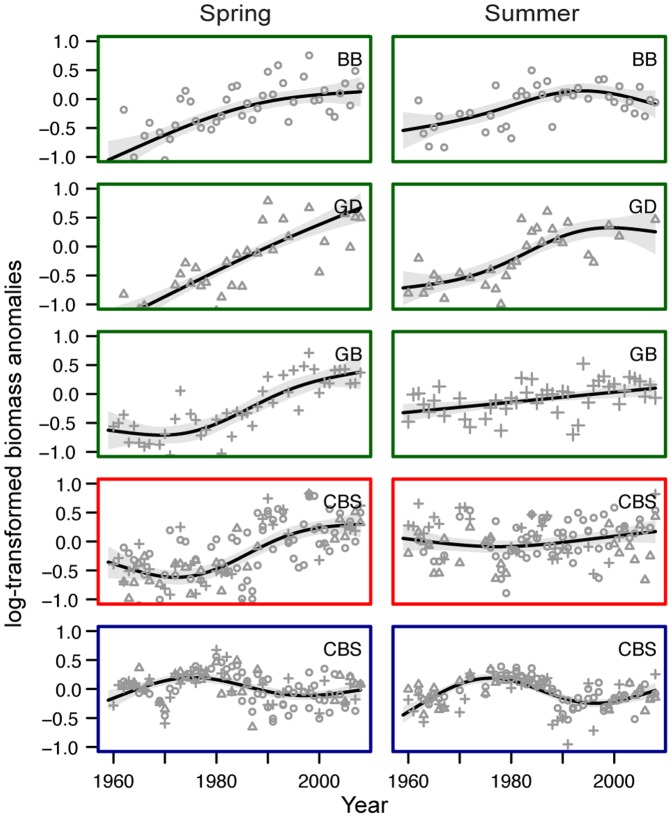
Observed and predicted long-term trends based on the best performing Generalized Additive Model (GAM). Spring and summer biomass anomalies of *Acartia* spp. (green boxes), significantly differed between the Bornholm Basin (BB), the Gdansk Deep (GD), and the Gotland Basin (GB), while trends for *Temora longicornis* (red boxes), and *Pseudocalanus acuspes* (blue boxes) were not basin-specific and rather consistent within the entire Central Baltic Sea (CBS) region. Open circles (BB), triangles (GD) or crosses (GB) represent the observed values in each basin, while the continuous lines indicate the predicted trends from the GAM based on basin-specific smoothers or a single smoother fore the entire CBS region. The shaded areas indicate the pointwise 95% CI.

The overall trend in *Acartia* spp. biomass was positive, particularly in spring in the Gdansk Deep (GD) where population sizes increased linearly with time (Fig. 2). This upward trend, however, leveled off at high biomass values at the end of the study period in the GD in summer as well as in both seasons in the Bornholm Basin (BB). In contrast, biomass in the Gotland Basin (GB) started to increase not until the late 1970s in spring with only a slight upwards trend in summer. *T. longicornis* biomass in spring increased as well, while in summer no significant trend was observed (Fig. 2, Table S3). *P. acuspes* biomass increased in all basins until the late 1970s, declined afterwards and increased again during the 2000s (Fig. 2).

### External forcing

**Figure 3:**
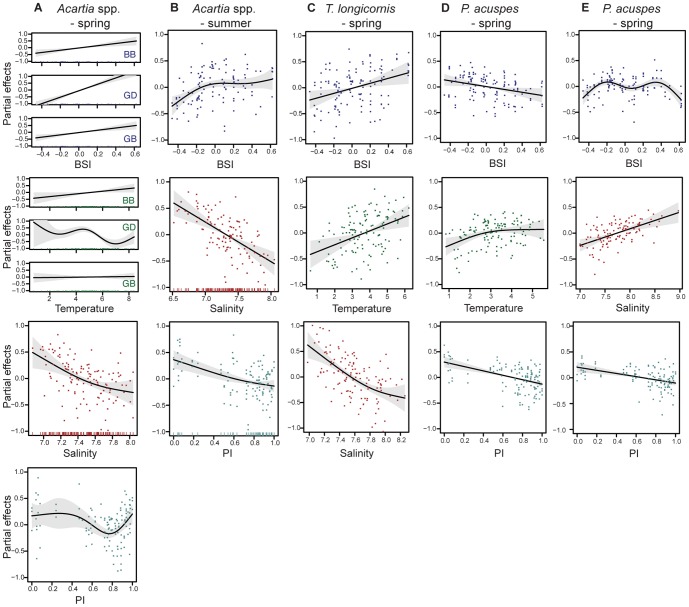
Statistical model results of *Acartia* spp., *T. longicornis*, and *P. acuspes*. Partial plots of significant covariates in the final spring and summer GAMs are presented for each basin separately or together depending on the significance and model performance. Values on the y-axis indicate the effect that the term on the x-axis has on the biomass anomaly. The solid lines indicate the smoothed (non-) parametric trend, shaded areas indicate the pointwise 95% CI.

Significant external forcing variables for long-term trends of our zooplankton indicator species were strongly species- and season-specific, and partly basin-specific (Table 2). The most parsimonious and best-fitting models for *Acartia* spp. spring and summer biomass explained 62% and 30% of the total variance, respectively. We found the climate index (BSI) to be the best predictor of annual variation in *Acartia* spp. biomass. For spring biomass the BSI effects were positive and linear, but of basin-specific strength (Fig. 3A). In contrast, the BSI effect on summer biomass was spatially consistent and non-linear with a stronger positive influence during periods with negative BSI anomalies (Fig. 3B). Temperature was a significant predictor of *Acartia* spp. biomass only in spring, but similar to the BSI of basin-specific strength. While the effect of temperature was weakly positive for the BB, it was highly significant and non-linear for the GD, with an overall negative trend.

**Table 2:** Summary of final Generalized Additive Models of species-specific responses to predation and hydro-climatic drivers in spring and summer.

*Acartia* spp. - Spring				*Acartia* spp. - Summer			
Covariate	edf	P-value	Expl. dev.	Covariate	edf	P-value	Expl. dev.
BSI : BB	1	0.003		BSI	2.745	<0.001	13.0%
BSI : GD	1	<0.001	39.3%	S	1	<0.001	7.24%
BSI : GB	1	0.001		PI	1.567	<0.001	7.21%
T : BB	1	0.049					
T : GD	3.78	<0.001	22.2%				
T : GB	1	0.779					
S	1.76	0.005	15.3%				
PI	3.25	0.002	19.5%				
R^2^ - adj. = 0.621				R^2^ - adj. = 0.299			

Temora longicornis - Spring							
Covariate	edf	P-value	Expl. dev.				
BSI	1	0.008	30.3%				
T	1	0.002	36.3%				
S	1.99	<0.001	19.6%				
R^2^ - adj. = 0.556							

Pseudocalanus acuspes - Spring				Pseudocalanus acuspes - Summer			
Covariate	edf	P-value	Expl. dev.	Covariate	edf	P-value	Expl. dev.
BSI	1	0.017	8.54%	BSI	3.891	<0.001	16.5%
T	2.04	0.01	8.18%	S	1	<0.001	23.1%
PI	1	<0.001	22.1%	PI	1.03	<0.001	40.4%
R^2^ - adj. = 0.275				R^2^ - adj. = 0.496			

Adjusted R^2^, estimated degrees of freedom (edf), significance (P-value), and individual explained deviance (excluding other significant effects) of the various covariates are provided. The covariate that explains most is indicated in bold. Note that for the *Acartia* spp. spring model the BSI and temperature smoother are presented for each basin separately but explained deviance is given for all three combined.

Covariates: BSI = Baltic Sea Index, T = Temperature, S = Salinity, PI = Predation Index.

Basins: BB = Bornholm Basin, GD = Gdansk Deep, GB = Gotland Basin.

Weaker predictors for *Acartia* spp. were salinity and predation, having uniform and generally negative linear effects in all basins. An exception represented the highly non-linear predation effect on spring biomass, being positive at the highest predation levels. As this is ecologically difficult to explain and rather points towards a spurious relationship, we conducted an alternative model selection excluding predation. This analysis resulted in significant autocorrelation, which we accounted for by including an exponential correlation structure. The selected alternative model contained only BSI as significant driver, but had less explanatory power than the model including predation (R^2^ = 0.29, Fig. S3).

Our statistical analyses for *T. longicornis* revealed no basin effects (Table 2). The most parsimonious and best-fitted spring model included, similarly to *Acartia* spp., a significant, albeit ecologically difficult to explain, predation effect (Fig. 3C). Hence we again applied an alternative model selection without predation, which resulted in the presented model, where most of the variance was explained by positive linear effects of temperature and BSI. Increasing salinity had additionally a strong, though negative influence. Differences between the models with and without predation were only minor, explaining 62% and 56% of the total variation, respectively. The variability in summer biomass, which lacked a clear trend, was only explained by the BSI. This driver showed a similar non-linear effect as with *Acartia* spp. but with limited explanatory power (explained only 5% of the fluctuations) and, thus, is not presented here.

Modeling long-term dynamics of *P. acuspes* revealed a particularly strong response to external drivers for summer biomass (the summer model explained 50% of the total variance) and no basin-specific relationships (Table 2, Fig. 3D–E). The most important driver was predation pressure with a negative effect explaining 22% and 40% in spring and summer biomass variation, respectively. Temperature had a generally positive and slightly non-linear effect on spring biomass while salinity had an overall positive effect on the summer biomass. The BSI showed a clear negative linear effect on total biomass in spring, although this effect might be predominantly on the nauplii [36] which, like *Acartia* spp., inhabit the surface waters. In summer, a highly non-linear BSI effect was observed with two difficult to explain maxima. Excluding this parameter would reduce the explanatory power of the model only by a few percent but would cause a strong impairment of the residual variance. We therefore kept the BSI in the final summer model.

## Discussion

### Effect of spatial habitat heterogeneity

Our findings suggest vertical habitat heterogeneity strongly determines the effect of climate variability on the three most important zooplankton species with considerable effects on overall food web functioning. *Acartia* spp. inhabit the surface water layers in the central Baltic Sea and changes in this group were mainly affected by changes in atmospheric conditions (i.e., BSI). This finding agrees with previous studies reporting that changes in zooplankton phenology and biomass levels were particularly sensitive to variability in climate-driven physical factors during the beginning of the productive season [25], [38], [43]. The strong BSI effect on *Acartia* spp. compared to spring temperature suggests that other factors related to the BSI are of importance, such as vertical mixing intensity affecting the timing of the spring bloom and phytoplankton development [37], [39]. Phytoplankton production is known to be an important driver for *Acartia* spp. [43], having a stronger effect on egg production than temperature [56], [57]. Although *Acartia* spp. shows maximum egg production rates at *Chl a* concentrations of > 14–20 µg L^−1^ [57] the species can also establish dense populations with much lower food concentrations (<2 µg *Chl a* L^−1^) [58], suggesting that not only food quantity (with *Chl a* as proxy) but also food quality can be important for growth and reproduction (see review in [59]). Unfortunately, we lack sufficiently resolved long-term phytoplankton data to investigate the effects of food quantity and quality as well as seasonal phenology on CBS zooplankton dynamics.

*Temora longicornis* inhabits the mid-water habitat and changes in the biomass of this species were influenced by both atmospheric conditions and water temperature. The positive temperature effect on the long-term spring dynamics has been described before [25], [38], whereas the relevance of primary production dynamics has only been shown recently [35]. Our data show that the long-term increase in spring biomass was, however, not translated to summer, as in *Acartia* spp., supporting the hypothesis that the increase reflects an earlier onset of population development rather than a numerical population response [35].

In contrast to *Acartia* spp. and *T. longicornis*, later developmental stages and adults of *Pseudocalanus acuspes* are directly exposed to fish predation in the deepwater habitat [31] explaining the statistical result of predation as the main driver of its long-term dynamics. The increase of the main predator sprat is due to increased temperature [60] and the decrease of its predator cod, the latter is partly due to lowered salinity and oxygen levels [61]. Hence, increased predation represents an indirect effect of climate [62]. *P. acuspes* showed also a strong positive response to increases in deepwater salinity in summer. This is in accordance with earlier studies claiming that this marine, glacial relict species prefers high salinities particularly for reproduction [25], [33], [63]. Younger stages (i.e., nauplii and copepodites C1-3) are highly abundant in spring, inhabiting the upper water layer [33]. Here they are affected mainly by temperature and other factors related to atmospheric forcing, such as phytoplankton dynamics [36].

In addition to the importance of vertical habitat heterogeneity for long-term dynamics of our study species, we were interested in the effect of spatial thermohaline variability on intra-specific population trends, which could have an effect on the community structure on a more regional scale. As this has not been adequately investigated in the realm of marine, pelagic systems, we compared long-term dynamics of populations of our study species that live in different basins of the CBS. We found the interaction of horizontal habitat heterogeneity with vertical habitat heterogeneity to be of importance. Although the CBS features a horizontal gradient in temperature and salinity, we did not observe basin-specific changes in biomass of *T. longicornis* or *P. acuspes*, which were strongly influenced by temperature and salinity respectively. A possible explanation is the temporal consistency of these gradients, as indicated by common long-term trends (Fig. S1). The lack of basin-specific responses indicates furthermore that differences in both hydrographic parameters seem not to be strong enough to cause basin-specific trajectories. For instance, instead of causing a much greater biomass decline of *P. acuspes* in the already less marine GB during the 1980s when salinity decreased, *P. acuspes* changed in the same way as in the BB, where salinity was always higher than in the GB (see Fig. S1). In contrast, *Acartia* spp. showed basin-specific long-term trends, coupled with spatial responses to changes in the BSI and temperature. The strong BSI effect on *Acartia* spp. biomass indicated that other factors such as wind mixing and consequently phytoplankton dynamics might be of importance. In spite of regional-scale atmospheric conditions wind-induced vertical circulation in the surface layers can show basin-specific patterns [64]. In line with this are the spatially differing long-term trends of different phytoplankton groups [39], [65]. The resultant changes in diet composition could have affected the lipid dynamics and stoichiometric regulation of *Acartia* spp. and consequently the energy available for growth and reproduction [45], [59].

### Role of climate in shaping the ecological niche space

The importance of habitat heterogeneity for community structure and dynamics is tightly related to the concept of the ecological niche. In general, habitats with more structure allow the coexistence of species with differing physiological requirements and environmental preferences [17], [66]. The range of a species' tolerance to the effects of multiple environmental factors was conceptualized by Hutchinson [67] as the n-dimensional hypervolume in environmental space that permits positive population growth, also termed as the fundamental niche (FN). This FN can also be understood as a fitness response-surface with an outer boundary defining the limit of population viability and inner contours representing increasing fitness [68]. Often, in the real world, not all combinations of environmental conditions, which could be favorable, are realized at a particular time or occur within a region. Consequently, only a subset of the FN exists which has been termed the potential niche (PN) by Jackson and Overpeck [69]. The PN may be substantially smaller than the FN [70]. The realized niche (RN) in this context represents the part of the PN that is constraint by biotic interactions (e.g., competition, predation) [67] or enlarged under high species dispersal [71], [72]. Hence, the RN can be envisioned as the populations’ response-surface in terms of, e.g., density or biomass [73]. When climate changes, the PN of a species can change in shape or size and similarly its RN due to additional changes in biotic interactions. Some species may benefit from these changes and increase in abundance, other species may persist but reduce in numbers, migrate or undergo local extinctions [74], [75]. Consequently, changes in community structure at a given locality depend not only on the magnitude of environmental changes but also on the level of species-specific niche differentiation.

In this respect, the increase in population size of *Acartia* spp. and *T. longicornis* can be interpreted as an improvement in the thermal space of their PN since both species showed a positive response to increasing spring temperatures. Both species further show highest egg production rates at similar temperatures, i.e., 13–18°C for *Acartia* spp. and 17°C for *T. longicornis*, and high mortalities at 24°C [32], [57], suggesting a great overlap in the optimal thermal space of their FN. Consequently, the PN of these two species in the upper 40 m of the CBS was likely sub-optimal in spring since water temperatures ranged generally only between 1 and 8°C (Fig. S1). A further warming would likely lead to increased productivity of these two species. This could be the case particularly for *T. longicornis* as this species responds stronger to temperature changes than *Acartia* spp. [76].

The role of biotic interactions such as predation becomes particularly apparent in the long-term dynamics of *P. acuspes*. Little is known about the response of its physiological processes to synergistic effects of environmental factors and, consequently, the size or shape of its FN or PN. But the fact that we identified predation as the most important factor for variation in the populations’ biomass suggests that mainly the RN space changed over time. For the marine copepod *Calanus finmarchicus*, Helaouët & Beaugrand [77] observed a strong coupling of the RN and the FN with rather similar niche widths. This congruency was explained by limited migration due to hydrodynamical barriers while biotic interactions were not discussed. For Baltic Sea *P. acuspes*, however, the RN is likely to be smaller owing to a strong top-down control through planktivorous fish. In general, the level of predation pressure can be reduced by behavioral adaptation. For instance, optical sampling has recently revealed predator avoidance behavior of *P.acuspes* females in the Baltic, which forces a high proportion of the population into sub-optimal habitats [78]. These habitats below the permanent halocline have low dissolved oxygen concentrations and are avoided in the absence of those predators. Such escapement behavior therefore only extends the outer boundary of the RN. In other words, the space in which the population is viable increases but not the space for optimum fitness [68].

### Conclusions

Our study findings suggest that habitat heterogeneity can modulate the response of zooplankton communities to climate changes and the extent in which the community structure can be altered. In the Baltic Sea, habitat heterogeneity has promoted a mixture of copepod species to exist in the central basins that have different fundamental niches and that display different sensitivities to climate-driven forcing. Consequently, the long-term development of these species differed greatly under changing environmental conditions, which in turn influences the community structure and dynamics. Individual habitats may also respond differently to climatic variability, as seen for the surface layers in the different basins, leading to differences in population trends even within the same species. We stress therefore the importance of understanding how anticipated climate change will affect ecological niches and habitats in order to project spatio-temporal changes in species abundance and distribution.

## Supporting Information

Figure S1 **Long-term trends of the hydrography within the Central Baltic Sea (CBS).** The main trend of the spring and summer temperature and salinity in different water layers (see Table S2) are given for each basin (red = Bornholm Basin BB, blue = Gdansk Deep GD, green = Gotland Basin GB) or the entire CBS region if trends and overall values were basin-unspecific (black line). Colored points represent the observed values in each basin, while the continuous lines indicate the predicted trends from Generalized Additive Models (GAM). Grey shaded areas indicate the associated standard errors.
(EPS)

Figure S2 **Time series of local climate and fish variability.** (A) Winter anomalies of the Baltic Sea Index (BSI) (Dec-March). (B) Predation index (PI) based on cod spawning stock biomass (SSB) (in black circles) in comparison to the shorter sprat SSB time series (in blue triangles). Both, BSI and PI have dimensionless units.
(EPS)

Figure S3 **Partial plot of the BSI effect in the alternative spring model of** ***Acartia*** **spp.** Values on the y-axis indicate the effect that the term on the x-axis has on the biomass anomaly. The number in parentheses on the y-axis indicates the estimated degrees of freedom. The solid line indicates the smoothed parametric trend, shaded areas indicate the pointwise 95% CI.
(EPS)

Table S1 **Overview of zooplankton datasets and their respective monitoring programs.**
(DOCX)

Table S2 **List of environmental variables used as covariates in the Generalized Additive Models (GAM) of at least one copepod species together with the definition and data source of each variable.**
(DOCX)

Table S3 **Summary of Generalized Additive Models (GAM) that performed best (based on** ***F*** **-ratio tests) in modeling seasonal biomass anomalies of each of our model species as a function of time.**
(DOCX)
