# Supplementary material for: Habitat Heterogeneity Determines Climate Impact on Zooplankton Community Structure and Dynamics
Source: PLoS One. 2014 Mar 10;9(3):e90875. doi: 10.1371/journal.pone.0090875 (PMC3948703; doi:10.1371/journal.pone.0090875)
Supplement: Table S1 — Overview of zooplankton datasets and their respective monitoring programs. (DOCX) [file pone.0090875.s004.docx]

**Table S1. Overview of the zooplankton datasets and their respective monitoring programs.**

| Data source | Spatial and temporal coverage | Gear type |
| --- | --- | --- |
| Latvian Institute of Food Safety, Animal Health and Environment (BIOR), Riga | Quarterly samplings of multiple stations (usually in February, May, August and October) were consistently conducted in the Eastern Gotland Basin since 1959. Stations in the Bornholm Basin and Gdansk Deep were sampled irregularly between 1959 and 1991. | A Juday Net (UNESCO 1979) with a mesh size of 160μm and an opening diameter of 0.36m. It is operated vertically and considered to quantitatively catch all copepodite stages as well as adult copepods, whereas nauplii may be slightly underestimated. Individual hauls were carried out in vertical steps, resulting in a full coverage of the water column to a maximum depth of 150m. |
| Finnish Environment Institute (SYKE), Helsinki | Sampling of one fixed station per basin in summer (mainly August) since 1979. While the sampling was conducted continuously until 2008 in the Gotland Basin, it stopped in 1990 in the Bornholm Basin and the Gdansk Deep. | A WP-2 with a mesh size of 100 μm hauled vertically in accordance with the Helsinki Commission (HELCOM) monitoring and assessment program (HELCOM 1988). |
| Leibniz-Institute for Baltic Sea Research (IOW), Warnemünde, Germany | Regular sampling (one to six times per season) of one fixed station in the Bornholm Basin since 1979. | A WP-2 with a mesh size of 100 μm hauled vertically in accordance with the Helsinki Commission (HELCOM) monitoring and assessment program (HELCOM 1988). |
