# Supplementary material for: Habitat Heterogeneity Determines Climate Impact on Zooplankton Community Structure and Dynamics
Source: PLoS One. 2014 Mar 10;9(3):e90875. doi: 10.1371/journal.pone.0090875 (PMC3948703; doi:10.1371/journal.pone.0090875)
Supplement: Table S2 — List of environmental variables used as covariates in the Generalized Additive Models (GAM) of at least one copepod species together with the definition and data source of each variable. (DOCX) [file pone.0090875.s005.docx]

**Table S2.** List of environmental variables used as covariates in the GAM analyses of at least one copepod species together with the definition and data source of each variable.

| Variable | Species | Definition | Data Source |
| --- | --- | --- | --- |
| surface salinity S1^spr^, S1^sum^ | A | mean salinity in spring (Apr-May) and summer (Jul-Aug); averaged for each basin (i.e. BB, GD, and GB) and the species-specific depth layer of 0-20m | bottle data from ICES database* |
| midwater salinity S2^spr^, S2^sum^ | T | mean salinity in spring (Apr-May) and summer (Jul-Aug); averaged for each basin (i.e. BB, GD, and GB) and the species-specific depth layer of 20-40m | bottle data from ICES database* |
| (mid- to) deepwater salinity S3^spr^, S3^sum^ | P | mean salinity in spring (Apr-May) and summer (Jul-Aug); averaged for each basin (i.e. BB, GD, and GB) and the species-specific depth layer of 30-50m in summer and the halocline region in spring (i.e. 60-90m in the BB, GD and 70-100m in the GB) | bottle data from ICES database* |
| surface temperature T1^spr^, T1^sum^ | A | mean temperature in spring (Apr-May) and summer (Jul-Aug); averaged for each basin (i.e. BB, GD, and GB) and the species-specific depth layer of 0-20m | bottle data from ICES database* |
| midwater temperature T2^spr^, T2^sum^ | T | mean temperature in spring (Apr-May) and summer (Jul-Aug); averaged for each basin (i.e. BB, GD, and GB) and the species-specific depth layer of 20-40m | bottle data from ICES database* |
| mid- to deepwater temperature T3^spr^, T3^sum^ | P | mean temperature in spring (Apr-May) and summer (Jul-Aug); averaged for each basin (i.e. BB, GD, and GB) and the species-specific depth layer of 20-60m | bottle data from ICES database* |
| PI |  | predation index based on Eastern Baltic cod spawning stock biomass (SSB); to mimic the predation pressure by planktivorous sprat we used inversely scaled values between 0 and 1. For this, we calculated the ratio between the difference of the annual SSB to the time series maximum and the overall min./max. range | VPA estimate, Eero et al. (2007) |
| BSI | A, T, P | Baltic Sea Index; mean value of winter months Dec-March. BSI calculations exist either based on sea level pressure values obtained from the NCEP (US National Centers for Environmental Prediction) or the SMHI (Swedish Meteorological and Hydrological Institute) data bank. Both time series differ slightly in their temporal coverage, causing deviations in the overall mean and consequently in the calculated monthly anamolies in overlapping years. In this study, the full time series (1970-2008) of the SMHI based BSI was used, since it is considered to better reflect the local situation (A. Lehmann, IFM-GEOMAR, pers. comm.) For the missing years 1960 – 1969, the index is based on NCEP data. | H.-H. Hinrichsen, GEOMAR Helmholtz Centre for Ocean Research Kiel, Germany |

A = *Acartia* ssp., T = *T. longicornis*, and P = *P. acuspes*

BB = Bornholm basin; GD = Gdansk Deep; GB = Gotland basin

* http://www.ices.dk/ocean/aspx/HydChem/HydChem.aspx

Eero M, Köster FW, Plikshs M, Thurow F (2007) Eastern Baltic cod (*Gadus morhua callarias)* stock dynamics: extending the analytical assessment back to the mid-1940s. ICES J Mar Sci 64: 1257-1271.
