# Supplementary material for: Habitat Heterogeneity Determines Climate Impact on Zooplankton Community Structure and Dynamics
Source: PLoS One. 2014 Mar 10;9(3):e90875. doi: 10.1371/journal.pone.0090875 (PMC3948703; doi:10.1371/journal.pone.0090875)
Supplement: Table S3 — Summary of Generalized Additive Models (GAM) that performed best (based on F -ratio tests) in modeling seasonal biomass anomalies of each of our model species as a function of time. (DOCX) [file pone.0090875.s006.docx]

**Table S3. Summary of Generalized Additive Models that performed best (based on *F*- ratio tests) in modelling seasonal biomass anomalies of each of our model species as a function of time**.

| ***Acartia* spp. - Spring** | | | ***Acartia* spp. - Summer** | | |
| --- | --- | --- | --- | --- | --- |
|  | Estimate | p-value |  | Estimate | p-value |
| Intercept | -0.26 | <0.001 | Intercept | -0.10 | <0.001 |
| Smooth term | edf | p-value | Smooth term | edf | p-value |
| *f_BB_*(Year) | 1.90 | <0.001 | *f_BB_*(Year) | 2.58 | <0.001 |
| *f_GD_*(Year) | 1.37 | <0.001 | *f_GD_*(Year) | 2.70 | <0.001 |
| *f_GB_*(Year) | 2.67 | <0.001 | *f_GB_*(Year) | 1.00 | 0.003 |
| R^2^=0.608 | | | R^2^ - adj. =0.42 | | |
| ***Temora longicornis* - Spring** | | | ***T. longicornis* - Summer** | | |
|  | Estimate | p-value |  | Estimate | p-value |
| Intercept | -0.21 | <0.001 | Intercept | -0.001 | 0.97 |
| Smooth term | edf | p-value | Smooth term | edf | p-value |
| *f*(Year) | 2.87 | <0.001 | *f*(Year) | 2.06 | 0.07 |
| R^2^ - adj. =0.417 | | | R^2^ - adj. =0.038 | | |
| ***Pseudocalanus acuspes* - Spring** | | | ***P. acuspes* - Summer** | | |
|  | Estimate | p-value |  | Estimate | p-value |
| Intercept | 0.02 | 0.27 | Intercept | -0.06 | 0.001 |
| Smooth term | edf | p-value | Smooth term | edf | p-value |
| *f*(Year) | 2.92 | <0.001 | *f*(Year) | 2.97 | <0.001 |
| R^2^ - adj. =0.163 | | | R^2^ - adj. =0.361 | | |

Intercept, smoothing term f and adjusted R2 are given. Smoothing terms with their respective effective degrees of freedom (edf) and p-values are presented either basin–specific or for the entire Central Baltic Sea.

BB = Bornholm Basin, GD = Gdansk Deep, GB = Gotland Basin
